# The relationship between lifestyle components and dietary patterns

**DOI:** 10.1017/S0029665120006898

**Published:** 2020-08

**Authors:** Andreea Gherasim, Lidia I. Arhire, Otilia Niță, Alina D. Popa, Mariana Graur, Laura Mihalache

**Affiliations:** 1 ‘Grigore T. Popa’ University of Medicine and Pharmacy, Faculty of Medicine, 16 Universității street, Iași 700115, Romania; 2 ‘Sf. Spiridon’ Clinical Emergency Hospital, 1 Independenței boulevard, Iași 700111, Romania

**Keywords:** Dietary pattern, Lifestyle, Dietary assessment

## Abstract

We conducted a narrative review on the interaction between dietary patterns with demographic and lifestyle variables in relation to health status assessment. The food pattern has the advantage of taking into account the correlations that may exist between foods or groups of foods, but also between nutrients. It is an alternative and complementary approach in analysing the relationship between nutrition and the risk of chronic diseases. For the determination of dietary patterns one can use indices/scores that evaluate the conformity of the diet with the nutrition guidelines or the established patterns (*a priori* approach). The methods more commonly used are based on exploratory data (*a posteriori*): cluster analysis and factor analysis. Dietary patterns may vary according to sex, socio-economic status, ethnicity, culture and other factors, but more, they may vary depending on different associations between these factors. The dietary pattern exerts its effects on health in a synergistic way or even in conjunction with other lifestyle factors, and we can therefore refer to a ‘pattern of lifestyle’.

Historically, nutritional literature has often reported on issues regarding the role of individual nutrient on health, but not all nutritional compounds in foods have been fully studied. The nutrient composition of foods varies considerably, and there are probably synergistic interactions between the nutritional components within any food, a topic that has been increasingly talked about lately^(1)^. Moreover, difficulties related to these interactions are also reflected in our present knowledge about the dietary patterns that people commonly consume. However, dietary patterns should be included in the development and implementation of nutritional guidelines, which could improve the chances of preventing non-communicable chronic diseases^(2)^.

It is worth mentioning that diet, as a lifestyle component, exerts its effects on health in a synergistic way or even in conjunction with other factors, which would not be reflected by examining each individual factor in isolation^(3–5)^. In nutritional research where only individual life factors were investigated, sophisticated statistical methods such as linear and logistic regression models have been selected to take into account the interaction and synergistic effects between these factors^(6,7)^.

The prevalence of chronic diseases increases as countries develop and become more industrialised. These diseases include obesity, high blood pressure, CVD, type-2 diabetes, neoplasms and many more. Dietary patterns play an important role in health and therefore in the prevention of chronic diseases^(8)^. Dietary patterns could as such provide a clearer, more accurate picture of a person's eating behaviour^(9)^. These models represent the interaction of all food choices that form a complete food pattern. These patterns are influenced by many factors, such as climate, demographics, religion, culture and others^(8)^.

The WHO considers diet to have the most important role in the prevention of chronic diseases and to be one of the most important lifestyle factors, emphasising the importance of understanding its complexity and its relationship with chronic diseases^(10)^. In addition to the unhealthy diet, the WHO identifies other important behavioural factors, such as physical inactivity, smoking and increased alcohol consumption as common risk factors for chronic diseases^(11,12)^.

Diet is one of the modifiable behaviours that can help reduce cardio-metabolic risk and prevent chronic diseases, so an assessment of the general dietary intake of the population becomes essential^(13)^.

However, it is imperative to investigate the lifestyle pattern as a whole, in order to better understand its implications on health and disease^(5)^. The aetiology of chronic diseases is complex and depends more on exposure to more overlapping/cumulative environmental factors rather than on exposure to a single factor, so the adoption of such holistic integration is encouraged. In fact, several prospective, or randomised, epidemiological studies have shown that these modifiable environmental factors, mentioned earlier, are all involved in the prevention and/or management of chronic diseases. There is also a lot of research on the association between each of these factors taken individually and in various chronic diseases. However, lifestyle factors most often exert their effects in a synergistic manner, a fact that would not be clear when studying each individual factor^(14)^.

Compared to the classical methods used in nutritional epidemiology, the approach of the lifestyle pattern confers a holistic representation in investigating the predisposing factors for the emergence of non-communicable chronic diseases^(15)^. Instead of examining a single factor (diet, physical activity, smoking, alcohol consumption and sleep) and its association with health/illness, this approach studies the entire lifestyle pattern and the interrelationships that may exist between these various lifestyle factors. As a result, a lifestyle pattern is distinguished as a dynamic interaction between factors, rather than emphasising each individual factor. Thus, the effects of a lifestyle pattern on cardio-metabolic health would outweigh those of its components taken individually (diet, physical activity, alcohol, smoking and sleep) and could thus detect more associations and implications in real life^(3)^.

Understanding dietary and lifestyle patterns would provide necessary evidence for planning intervention and education strategies^(16)^.

## Assessment of dietary intake

Of all the available subjective methods that evaluate a person's nutritional intake: 24-h recall, food record, food history and FFQ, the last has been the most widely used in epidemiological research. Nutritional data have been obtained either by a trained interviewer or through self-reporting^(17)^.

FFQ is the most appropriate dietary assessment tool because it is easy to apply by the researcher. The method is actually an advanced form of food history. It has two components: a qualitative one that investigates the frequency of consumption of a food, and a quantitative one that estimates the amount of food consumed with the aid of a photographic atlas or using some culinary measures. The subjects answer on how often and how much food they have consumed in a given period of time^(18)^. FFQ can focus on the intake of specific nutrients^(19)^, dietary exposure to a particular group of foods only (which may be linked to a particular disease)^(20)^ or on assessing the inter-correlations between nutrients and between foods (i.e. the dietary pattern) with their effects on health status/risk of disease^(21,22)^.

In addition to nutritional inquiry, particular biochemical markers have been used to measure the dietary intake of specific nutrients or foods^(23–25)^. Nutritional biomarkers offer objective estimates of dietary exposure in anthropometric and clinical assessment, while 24-h recall, food record, food history and FFQ are subjective estimates^(17)^. However, some biomarkers may be affected by disease or physiologically, by homoeostatic regulation, so they cannot provide information on the absolute dietary intake of the subject. In addition, dietary recommendations aimed to change the eating habits of a subject cannot be made solely on the basis of biomarkers^(26)^. Thus, direct assessment of food intake through surveys can sometimes be more informative than biomarkers^(27)^.

However, in order to obtain the most accurate estimates of food intake, it has been proposed that a combination of methods, such as FFQ with 24-h recall or FFQ with biomarkers, should be used, instead of individual method^(17)^.

## Dietary pattern methodology

FFQ is a reliable and inexpensive data collection method, which allows the identification and evaluation of food patterns in epidemiological studies^(28)^. Although dietary patterns based on the 24-h recall do not most accurately assess an individual's usual eating patterns, it is also a widely accepted method for evaluating food intake at the population level^(29)^.

Food pattern analysis has the advantage of taking into account the correlations that may exist between certain foods or groups of foods, but also between nutrients^(30)^. It represents a broader picture of dietary intake, analysing the effects of diet as a whole. It is an alternative and supportive approach to analysing the relationship between nutrition and the risk of chronic diseases, thus being more predictive of the risk of disease compared to individual foods or individual nutrients^(31)^.

Two different statistical approaches have been used in the literature for analysing dietary patterns^(32)^:

(1) *a priori* or theoretical approach which consists of defining certain quality scores or indices of the diet based on nutritional recommendations, for different categories of subjects^(33,34)^;
(2) *a posteriori* approach which is defined by the use of methods based on exploratory data:
  (i) dietary patterns based on variations in food intake, using factorial analysis (principal component analysis) or cluster analysis^(35,36)^
  (ii) dietary patterns based on a combination of exploratory data and *a priori* knowledge about a disease (information on the pathological mechanisms of the diseases in relation to certain food models), identified using the low-rank regression^(37,38)^.

Both factor analysis and cluster analysis are considered *a posteriori* because food models are obtained by statistical modelling of dietary data^(39)^. One of the *a posteriori* approaches is to derive food patterns based on the variation in specific markers related to health/disease^(40,41)^. However, there are some limitations, in the sense that if one cannot take into account the daily variation of dietary intake at the individual level, the statistical power in detecting the correct and real associations between the dietary patterns and certain diseases can be reduced^(42)^. Because *a posteriori* approaches generate patterns based on available empirical data, but which do not have an *a priori* hypothesis, they do not necessarily represent optimal models. Conversely, the approach of the quality index also presents some limitations (present knowledge and the ability to understand the diet–disease relationship), as well as uncertainties in selecting the individual components for the composition of the score and uncertainties related to the subjectivity in defining some cutoff points^(32)^.

Although these approaches are based on different methods, all of them can help identify healthy or unhealthy dietary patterns and can be the basis for developing and implementing nutritional guidelines^(2)^.

### *A priori* methods

These methods use nutritional variables that are quantified in such a way that they could provide an overall assessment of diet quality, thus being important for the definition of health status^(43)^.

*A priori* approaches based on scores are constructed according to certain dietary guidelines and include selected nutrients and/or foods and/or food groups, according to nutritional recommendations, thus establishing a certain score. Later, the data are grouped, referring to the predefined ones, to obtain a score (a measure of diet quality)^(44)^.

The diet quality index (DQI)^(45)^ is a score of the degree to which an individual's diet is in accordance with the specific dietary recommendations. DQI are tools that aim to assess the quality of diets and thus allow individuals to be classified based on the degree to which they have a ‘healthy’ eating behaviour^(33)^. Individuals are scored for each component, then a score is calculated for each individual; high scores reflect dietary intake according to nutritional recommendations^(46)^.

Another simple and common score is the dietary diversity score, which takes into account the number of portions in the food groups (i.e. dairy, meat, cereals, fruits and vegetables) or foods consumed regularly^(47,48)^.

Presently, there are a large number of DQI, most of them being designed, defined or adapted to express the nutritional needs of different population groups and to highlight the compliance to specific food patterns^(49)^.

There are three major categories of indicators:

(1) nutrient-based indicators, which need to be transformed from quantity (weight of food) to quality (nutrient content), and subsequently compared with standard requirements^(33)^;
(2) food/food group-based indicators that use food guides to assess portions, frequencies or food groups^(50)^;
(3) a combination of indicators, which refer to dietary variety within and between food groups, to adequate nutrient intake (compared to standard recommendations) or to suitable intake of food groups (quantities or portions), to the frequency of food consumption, and also to a general balance of macronutrients^(51)^.

DQI use a scoring system, which can establish adherence to a dietary model defined *a priori* and can thus be used to measure the quality of diets within a population. The best example is the healthy eating index. This is a DQI, created and validated in 1995 by the US Department of Agriculture, to reflect the nutritional recommendations in the Dietary Guidelines for Americans^(51)^.

The analysis of the updated healthy eating index-2015 captures the variation of diet quality in a way that takes into account the multivariate nature of healthy diets. This method of evaluation, the updated index, captures some elements of real interest: high-quality diets have high scores, scores vary within the population, diet quality varies between different groups of people, diet quality is evaluated independently of quantity, diet quality is multidimensional, distinct dietary components can be captured, and not least, it is associated with a reduced risk of general mortality and morbidity, showing the validity of the criterion^(52)^.

The main advantage of the *a priori* method is its generalisation (it can be applied to several populations)^(44)^. Each DQI has different food and nutrient components, as well as different approaches to score, which makes comparability limited. Moreover, most DQI have been developed only for certain specific populations and cannot be widely used in others. Also, it is difficult to compare the results between studies using different DQI^(53)^. The subjectivity of the investigator responsible for the definition of indices, and the fact that they are based on nutritional recommendations, which have been defined for certain populations, thus not making them possible to be applicable to others^(44)^, could be major disadvantages when analysing data.

### *A posteriori* methods

The study of nutritional patterns using data extraction methods, based on correlations between food groups, has been proposed in nutritional epidemiology by most researchers^(8)^. For this purpose the statistical techniques used are: factor analysis, cluster analysis and low rank regression^(44)^.

Factor analysis (principal component analysis) is a multivariate statistical technique, which uses information obtained from FFQ to identify food consumption factors (or patterns)^(39)^. It aggregates and reduces the dietary data to a correlation between foods, which would explain the greatest variation in the diet of the studied group^(54)^. A score is obtained for each model (factor and dietary pattern), which can be used subsequently, by statistical methods of correlation or regression, to examine relationships, such as, for example, nutrient intake, in association with cardiovascular risk factors and other biochemical indicators of health^(39)^. The use of factor analysis to identify food patterns may have limitations. The results of factor analysis can be affected by subjective, but important, decisions that need to be taken in defining the food pattern: the allocation of food in food groups, which variables to be included in the analysis to build the patterns, which variables can contribute to the definition of a factor, the number of extraction factors, the rotation method and even the labelling of factors (the name of the pattern). Such decisions can lead to erroneous conclusions^(44)^. However, this method offers the opportunity to summarise and refines the data to a simpler descriptive model^(55)^.

Principal component analysis has a long-term reproducibility, stability and validity compared to other methods^(56)^ that could minimise the risk of errors. Unlike cluster analysis that involves empirical classification, principal component analysis theoretically establishes a causal relationship between indicators (items). It does not describe the natural patterns of the population in the study, but explains the important variation within the population^(56)^.

However, exploratory analysis is used when there is an *a priori* hypothesis about the factor structure. As such, its advantage is that it reduces some of the subjectivity associated with the exploration procedure and can be applied in different population samples^(44)^.

Using factor analysis, most researchers identified two major patterns. The first pattern, labelled ‘prudent’, is in general characterised by a higher intake of vegetables, fruit, legumes, whole grains and fish, while the second pattern, labelled ‘western’, is characterised by a higher contribution of processed meat, red meat, butter, high fat dairy products, eggs and refined grains. The main patterns identified by factor analyses are in accordance with the *a priori* knowledge^(32)^. Another common pattern identified in European studies is the Mediterranean one, characterised by increased intake of vegetables, fruit, fish and olive oil^(22)^. Moreover, this statistical methodology has been extended to other regions of the world, making it possible to identify another pattern, the traditional one. For example, the traditional Japanese pattern^(57)^, characterised by consumption of vegetables, seafood, soya, fish, fruit, green tea, miso soup; or the traditional Brazilian pattern^(58)^, characterised by the consumption of white rice, grains, beer, fresh and processed meat.

Cluster analysis is another multivariate statistical method that allows the characterisation of food patterns. Unlike factor analysis, cluster analysis aggregates individuals into relatively homogeneous subgroups that have similar diets. Individuals can be classified into separate groups or similar groups based on the frequency of food consumed, the percentage of energy contributed by each food or food group, the average amount of food intake (g), nutrient intake or a combination of dietary and biochemical measures^(39)^. A certain amount of subjectivity is also included in this method: the choice of variables to be included in the analysis and the number of factors to be included or at what level of significance to apply the variables^(44)^.

The RRR (reduced rank regression) statistical method combines two sources of information (preliminary data and study data). RRR reduces the size of predictor variables (e.g. food intake or specific food groups) to the size of response variables (e.g. nutrients as biomarkers)^(59)^. RRR analysis produces a linear combination of food groups that explains the maximum variation of response variables^(60)^. RRR identifies linear functions of predictors that explain as many response variations as possible^(61)^. As with other *a posteriori* approaches, it is difficult to assess whether the food model can be applied to different groups of populations, and to compare the results later^(60)^.

The results obtained by either of these methods indicate which food/beverage combination best predicts the health/disease condition. However, this approach itself does not take into account other non-dietary variables and thus cannot provide information on whether these associations between dietary pattern and health/disease persist after adjusting for socio-demographic or other lifestyle factors^(37)^.

## Other lifestyle components in relation to dietary patterns

From an epidemiological point of view, diet is a complex combination of exposures. However, experimental epidemiological studies often fail to certify the effects observed for all dietary components. The conventional approach adopted in food consumption investigations is focused on assessing the intake of energy, nutrients or foods as independent variables, and this does not take into account the effect of the diet as a whole on risk diseases, and confusions and interactions that may occur between different dietary components are not properly considered^(62,63)^.

Dietary patterns are supposed to illustrate the real situation of dietary availability and dietary practices of the studied population^(64)^. As a result, they facilitate the identification of subgroups that adopt dietary habits that are compatible with risk or protection against chronic diseases and provide credible scientific support for developing dietary guidelines^(65)^.

Diet is a major modifiable determinant of most chronic diseases. It is known that dietary choices are strongly influenced by socio-demographic determinants and other lifestyle factors. Therefore, identifying the determinants of food consumption is essential for examining their possible contribution to the prevalence of the disease^(66)^.

Indeed, several individual factors have been shown to be associated with food patterns^(9)^. Both healthy and unhealthy dietary patterns may vary according to sex, socio-economic status, ethnicity, culture and other factors^(32,67)^, but moreover, they may vary depending on different associations between these factors. In recent years, researchers have tended to group lifestyle factors. These risk factors are not arbitrarily spread in the population, but they appear in combination with other lifestyle risk factors. The grouping of the risk factors of the lifestyle is related to a higher degree of association with different diseases than we would expect from each of the individual risk factors. Because lifestyle groups in a community can be associated with different patterns of demographic and social risk factors^(68)^, identifying different lifestyle patterns and associated factors across the country may be helpful in finding high risk subgroups, which require appropriate interventions^(69)^. For example, age and education are positively associated with a healthy diet (characterised mainly by high intake of fruit, vegetables or fish)^(70)^. In addition, the connection between physical inactivity, smoking and young age was associated with an unhealthy diet^(71)^. Healthy diet (and its major healthy dietary components), moderate alcohol consumption, non-smoking status, normal weight and regular physical activity have been associated with a lower risk of premature mortality and a longer life expectancy^(72)^. Moreover, there is much evidence that stresses the role of nutrition, in relation to that of physical activity and sleep, on health and mortality^(73,74)^.

The health consequences of adopting unhealthy lifestyle habits cannot be overestimated and, therefore, specific policy strategies or an appropriate action plan are needed to reduce adhering to an unhealthy diet and/or promote healthy diets, to reduce physical inactivity and/or promote physical activity or to reduce the increased consumption of alcohol and tobacco^(16)^. Given the potential for synergistic relationships between diet, physical activity, sleep, concomitant improvement of multiple lifestyle behaviours may have the potential to deliver greater health benefits compared to single behavioural improvement^(75)^. Thus, identifying groups with healthier eating patterns would allow better nutritional strategies in terms of population nutrition^(56)^.

### Sex

In the literature, it is commonly found that women generally have higher scores for the pattern considered healthy and lower scores for the pattern considered unhealthy^(76)^, while men are usually associated with unhealthier patterns (Western, Western-like or others, which are characterised by high fat, meat or fast-food intake)^(77,78)^.

### Age

Several socio-demographic characteristics and family lifestyle were related to the child's eating patterns^(79)^ and the nutritional status of the child^(80)^. These factors are thus important to consider when studying the relationship between diet and weight status.

During childhood and adolescence, the evaluation and monitoring of food intake and other health behaviours is particularly important^(81)^, as these are decisive steps in forming eating habits and maintaining them into adulthood^(82)^. In recent years, due to the increased prevalence of overweight worldwide, the need to monitor eating habits among young people has increased^(83)^. One method that helps nutritional assessment in children/adolescents is the development of particular online nutritional surveillance systems, designed to collect periodic information on weight status (based on BMI), food consumption, hours of physical activity or sedentary behaviour, food consumption at school meals. Such data allow the following of anthropometric parameters, food patterns and other healthy/unhealthy behaviours, as well as their association with weight status^(81,84)^.

Changes in daily patterns, such as daily school hours or the weekends, clearly and significantly contribute to changes in food intake, to a pattern of physical activity, and ultimately to energy balance^(85)^. In children, a so-called ‘traditional’ pattern was identified, characterised by a consumption of certain foods reported on school days, i.e. on weekdays (different from food consumption on non-school days, weekends or holidays). Food quality was found to be lower at the end of the week compared to the weekdays, with a significantly higher intake of total sugars^(86)^, sweetened beverages, confectionery, pastry, snacks and at the same time, with lower consumption of fruit and vegetables^(87)^. Therefore, school meals seem to play a particularly important role in promoting healthy eating, by creating opportunities and benefits for expanding the diversity of food groups and establishing a benchmark for healthy eating^(81)^.

Exploratory data-based methodologies to examine the interrelationships between eating patterns, physical activity and sedentary behaviours in children and adolescents have shown that healthy and unhealthy patterns are grouped in a variety of ways that are both beneficial and harmful to health^(88)^. Thus, one can talk about the ‘mixed’ eating pattern, which is characterised by the presence of both healthy and unhealthy foods^(81)^.

A multitude of internal and external factors can influence adolescents' eating patterns. Internal factors include: self-image, physiological needs, individual health, values, preferences and psychosocial development. And among the external factors, there are mentioned: family habits, friendships, social and cultural values and rules, the media, individual tendencies, personal experience and knowledge^(89)^.

The nutritional status of adolescents is the result of interrelated factors, influenced by the quality and quantity of food consumed and by the physical health of the individual and has important implications for their health, thereby playing a key role in the development/prevention of several chronic diseases. During adolescence, changes in an individual's lifestyle may affect eating habits and choices, but there are also physical changes that affect the nutritional needs of the body^(90)^.

One of the most common patterns among adolescents includes: snacks (usually high-energy foods), lack of a main meal (especially breakfast) or irregular meals, the predominance of fast-foods, with reduced consumption of fruit and vegetables^(90)^. Thus, unhealthy eating patterns among young people could promote the prevalence of obesity and cardiovascular risk factors in this population group^(91)^. It is evident from epidemiological research that the pattern characterised by intake of meat and French fries has been associated with an increase in the prevalence of diabetes^(92)^ and acute myocardial infarction, while a pattern characterised by fruit and vegetables has been proven to be protective^(93)^.

Regarding older subjects, the literature found that they usually have a higher adherence to the fruit and vegetable patterns and are more likely to consume foods included in the healthy eating index^(94)^ and a lower adherence to the meat and French fries pattern^(76)^. However, also among the elderly, there was also a high score for the pattern with high fat and sugar intake. This could be due to several factors, including hypogeusia^(95)^ or a decrease in financial capacity (which forces older people to buy less expensive, sweeter or high-fat foods)^(96)^. Important components of the ageing process are included in the healthy eating pattern, as they contain nutrients that protect against systemic inflammation and endothelial dysfunction^(97)^. Adopting this pattern would delay the onset of age-related diseases^(98)^. The association between a healthy eating pattern and adherence to at least two other factors of a healthy lifestyle has been shown to be correlated with decreased mortality in the elderly^(99)^.

### Socio-economic determinants

Several variables (such as education, income, type of employment and some characteristics of the particular areas in which populations live) that characterise the socio-economic status of different populations around the world are closely linked to diet quality. However, there is no unanimity on how education or income levels affect diet quality^(53)^.

Very complex interactions between education, income level and occupation are identified. A low level of education, a low income or a low professional position were associated with an unhealthy dietary pattern^(7)^ and with low quality diet, characterised by lower fibre, mineral and vitamin intake^(100)^, while a higher educational level or higher professional position were associated with a healthy dietary pattern^(101)^.

Although not generally valid, in most cases the urban environment is associated with healthy eating patterns, which include greater dietary diversity and yet with a food intake of animal origin. In contrast, rural dwellers from low-income countries, and even some middle-income countries, still rely on unhealthy food preservation methods (e.g. salting or smoking)^(102)^.

The relationships between diet quality and socioeconomic status internally are important to evaluate, as diet quality can be influenced by other factors (target population, unemployment, occupation of different family members, access to food, urbanisation in countries with small or large gross domestic products)^(103)^. Unhealthy behaviours tend to be present, especially in people with low socio-economic status^(67)^.

Highly educated participants had higher scores for fruit and vegetables and lower scores for fried meat and potatoes and to a lesser extent for fat and sugar models, a finding repeatedly reported in the literature^(76)^. One likely explanation is that the dietary intake of highly educated persons is in line with dietary recommendations^(104)^. These people tend to have higher incomes that allow them to buy more fruit and vegetables than less educated people^(96)^.

Lower education has been associated with unhealthy eating patterns. Cheaper, unhealthier, high-risk foods for chronic diseases have been found more often especially among low-educated women^(105)^.

### Students

The Western lifestyle has led to changes in eating habits among young students in developing countries. The populations of university students are characterised by physical inactivity, sedentary behaviours and unhealthy dietary behaviours, i.e. irregular meals, inadequate snacks, high consumption of fast food and insufficient consumption of fruit and vegetables^(106)^. To enable them to cope with the energy needs of the body, as they carry out their normal academic activities^(90)^, most students consume frequent snacks outside the main meals. Low levels of physical activity and unhealthy eating patterns are not compatible with national recommendations for a healthy active lifestyle for young people and may contribute to increasing the rate of overweight and obesity in this population^(107)^, and therefore individuals are more prone to developing type-2 diabetes mellitus and CVD^(108,109)^.

### Smoking

There is extensive information on the relationship between nutrient intake and smoking; smoking is associated with less healthy eating behaviour, regardless of culture, ethnicity or region^(110)^. Smoking is associated with both reduced antioxidant intake and increased turnover of these micronutrients^(111)^. Smokers usually have lower scores for the prudent model^(112)^. Present smoking selectively affects the consumption of specific foods. Possible explanations include non-adherence to dietary recommendations^(104)^, as well as tobacco-induced changes in the sensory system, taste impairment^(113)^ and decreased olfactory capacity^(114)^, causing smokers to select foods with stronger/saltier/unhealthier flavours^(9)^.

Compared to non-smokers, smokers are more likely to adopt an unhealthy dietary pattern if they have a low educational level, but a lower probability of such pattern if they have a high educational level^(67)^. A low level of education, in combination with physical inactivity and smoking were linked also to a lower adherence to a Mediterranean-style diet^(115)^.

### Alcohol

Moderate alcohol intake is an important component of the Mediterranean pattern^(116)^, which has been shown to be a protective factor against cardiovascular mortality, myocardial infarction or stroke^(117)^. Those who consume wine in moderation usually have healthier lifestyles than other types of alcohol consumers, smoke less and take more physical activity, with increased fruit and vegetable consumption and reduced red meat and fried foods^(118)^.

Increased alcohol consumption has been associated with the risk of hypertension^(16)^ and stroke^(119)^. Those who consume alcohol in high amounts tend to have associated unhealthy behaviours, such as poor quality diets, low physical activity and a general tendency for reckless actions that lead to an increased risk of mortality^(120)^.

### Previous dieting

The literature reports that those who have followed different diets over time have higher scores for the prudent model and lower scores for models characterised by meat, fries, fats and sugar, possibly due to increased awareness of the importance of food intake^(121)^. Due to the large variation in the type of diet, it is not possible to accurately assess the associations between each type of previous diet and the presently different dietary patterns^(9)^.

### Sedentariness

Sedentary lifestyle is associated with a low adherence to the fruit/vegetable pattern and has the tendency to be associated with a higher score for the unhealthy pattern (animal fats and sweets). Such findings have been repeatedly reported in the literature^(112)^. Food patterns are closely linked to several lifestyle features^(9)^, which relate to sedentary behaviours, including watching TV. These behaviours were related to high consumption of sweetened beverages, ready-made products, sweet foods, snacks, fast food and alcohol^(122)^.

### Obesity

People with obesity may underestimate the intake of foods they consider to cause obesity. In general, increased BMI is associated with unhealthy dietary patterns^(9)^. The anthropometric parameters are related to the model ‘fats and sugar, meat and fries’. This association may be due to the fact that most people do not consider meat as obesogenic^(123)^. In addition, a better BMI is associated with the ‘fruit and vegetables’ pattern^(124)^. Thus, frequent consumption of fruit and vegetables, while respecting restrictions on the amount of food consumed and at least moderate physical activity during leisure, are associated with a lower probability of overweight/obesity^(125)^.

### Sleep

Sleep disorders, including short sleep duration, are recognised as a risk factor for the negative outcomes of an unhealthy lifestyle^(126)^. Sleep is a key modulator of metabolic functioning, including energy metabolism, glucose regulation and appetite^(127)^; research conducted in recent years has focused on the effects of sleep duration and dietary intake, especially as sleep may present a modifiable risk factor for chronic non-communicable diseases, such as obesity^(128)^. Short sleep duration is associated with a lower variety of foods and thus a lower intake of protein, carbohydrates, fibre and fat compared to normal sleep duration^(129)^. In particular, total serum carotenoid concentrations were associated with a higher probability of short sleep duration (5–6 h per night) compared to normal sleep duration (7–8 h per night)^(130)^.

The relationship between sleep and diet quality is bidirectional^(131)^. Sleep has an impact on diet, but conversely, diet/specific foods/dietary patterns have an impact on sleep^(128)^. Short sleep duration is associated with weight gain through effects on appetite, physical activity and/or thermoregulation^(132)^. An inverse association between sleep duration and BMI is described, however, long sleep on weekdays was associated with a lower score of healthy eating pattern compared to normal sleep duration^(133)^. Another important consideration is not only the types of foods, but also regular meals, as well as the last meal at which these foods are consumed, which may be important for sleep^(131)^.

### Transitions

The literature shows that dietary habits change over time (in the same individual or at the population level), and this helps us understand how changes in eating pattern are reflected in the health status^(28)^. Diet and dietary pattern can undergo drastic changes during transitional periods: from a single person to a married person, or during certain significant events in the marital sphere (i.e. death and divorce), which have been shown to have repercussions on food consumption^(134)^. Thus, further research should focus on and address changes in dietary patterns throughout life (with a greater focus on important life transitions).

Population movement within the same countries, from rural to urban areas, may also be related to these changes in diets, frequently to some healthier models^(135)^. The linguistic region of a country is another major determinant of patterns, which have a particular distribution among the linguistic regions, apparently reflecting the cultural influence of the respective neighbouring countries on the food patterns^(66)^.

When it comes to relationships, women in a couple are more likely to adhere to a healthy eating pattern, compared to single women, who are less likely to follow dietary recommendations^(136)^.

During pregnancy, dietary composition can play an important role in pregnancy and fetal weight^(137)^. During pregnancy it is essential that the dietary pattern be prudent, which provides the energetic and nutritional intake necessary for maternal health, so as to contribute to the prevention of pregnancy-related diseases and to allow for fetal growth and development under favourable conditions. The nutritional status of the mother during the preconception and/or during pregnancy may affect the perinatal phase of the pregnancy outcome^(138)^. Higher weekly weight gain is linked to greater adherence to a dietary pattern characterised by high intake of sweets, fast foods and snacks, while a pattern characterised by increased intake of vegetables, fruit and fish was not associated with gestational weight gain^(139)^. Also, it was observed that the patterns do not change significantly over time. Therefore, a correct assessment of the food intake obtained at any given time during pregnancy can provide basic information about the dietary pattern throughout the pregnancy^(140)^.

### Breakfast

There is scientific evidence to suggest that the pattern of a meal is an important determinant of diet quality, energy consumption and nutrient content and, thus, cardio-metabolic health^(141,142)^. For example, skipping breakfast is associated with poor diet quality^(143)^ and thus with adverse cardio-metabolic health outcome^(144)^. The nutritional composition of breakfast should also be taken into account when this meal is present^(145)^, as is the pattern of the other meals throughout the day^(142)^.

In adults it has been shown that daily breakfast intake improves the intake of nutrients, the selection of food groups and therefore the quality of diet^(143,146)^. In general, breakfast consumption is associated with improved adiposity parameters^(147)^, decreased cardiovascular risk factors^(148)^ or decreased risk of adverse effects related to glucose and insulin metabolism. Breakfast can contribute to a healthier diet, which can also lead to cardio-metabolic improvements^(149)^. Breakfast skipping is a very common practice among students and, despite this fact, and even if the consumption of certain food groups is avoided, the proportion of young people with overweight and obesity tends to increase^(150)^.

### Late meal in the evening/at night

Several cross-sectional studies have shown an association between late night food intake, in combination with skipped breakfast, and a higher risk of adverse health effects^(149)^, including metabolic syndrome^(151)^.

This particular form of the late-meal pattern is present more frequently in young adults and students^(152,153)^ and refers to the chronological type, i.e. the individual preferences for sleep time and eating behaviour; morning or evening type^(154)^. In terms of eating behaviour, studies show that evening types associate less healthy eating habits (main meal later in the day both on work- and non-working days, a tendency towards fewer meals daily, with larger portions, higher energetic intake and inadequate vitamins and minerals) and have a higher BMI^(154,155)^. Night time eating, in particular, has been identified as a risk factor for metabolic syndrome and obesity^(156,157)^.

The late evening meal was found to be associated with sleep apnoea, with lower levels of HDL-cholesterol and higher levels of stress hormones^(154)^. Night meals are also associated with a higher risk of obesity^(158)^.

### Occasional/out-of-home meals

Neutral terms ‘occasionally eaten’ or ‘eaten at an event’ or ‘outside the home’ are used to describe any occasion where food or drink is consumed and therefore includes all types of foods. Meals are described taking into account: modelling (e.g. frequency, regularity, irregularity, spacing and timing), format (e.g. different food combinations and nutritional content) and context (e.g. eating together with others or with the family, eating meals in front of the television or outside the house)^(142)^.

Regarding sex, it is known that men consume more meals that are not prepared at home than women^(16)^. Meals prepared outside the house, especially fast foods, contain high levels of energy and a low amount of nutritional compounds^(159)^. Therefore, the frequency of food consumption in restaurants is positively associated with the increase in body fat in adults. In general, people with obesity choose a larger quantity of food in the restaurant than the normal-weight people^(160)^. Conversely, frequent consumption of home-cooked meals is associated with a lower risk of developing cardio-metabolic disease, such as diabetes^(161)^ or obesity^(162)^.

## Conclusions

The complex interconnections between nutrients, foods and dietary patterns imply that no individual component of the diet can provide a complete picture of the favourable/unfavourable effects of diet on health, thus a methodical approach using evidence-based on dietary patterns is warranted.

It is clear that lifestyle, of which an important component is the diet, is of great importance for health. The dietary pattern exerts its effects on health in a synergistic way or even in conjunction with other lifestyle environmental factors, and we can therefore acknowledge the role of a ‘healthy lifestyle pattern’.

## References

[1] Jacobs DR & Tapsell LC (2007) Food, not nutrients, is the fundamental unit in nutrition. Nutr Rev 65, 439–450. 17972438 10.1111/j.1753-4887.2007.tb00269.x

[2] Tapsell LC, Neale EP, Satija A (2016) Foods, nutrients, and dietary patterns: interconnections and implications for dietary guidelines. Adv Nutr 7, 445–454. 27184272 10.3945/an.115.011718 PMC4863273

[3] Al Thani M, Al Thani AA, Al-Chetachi W (2016) A ‘high risk’ lifestyle pattern is associated with metabolic syndrome among Qatari women of reproductive age: a cross-sectional study. Int J Mol Sci 17, 698. 10.3390/ijms17060698 PMC4926323 27271596

[4] Steele EM, Claro RM & Monteiro CA (2014) Behavioural patterns of protective and risk factors for non-communicable diseases in Brazil. Public Health Nutr 17, 369–375. 23308392 10.1017/S1368980012005472 PMC10282382

[5] Naja F, Itani L, Nasrallah MP A healthy lifestyle pattern is associated with a metabolically healthy phenotype in overweight and obese adults: a cross–sectional study. Eur J Nutr [Epublication 30 July 2019]. 10.1007/s00394-019-02063-9 31363827

[6] Hankinson AL, Daviglus ML, Horn LV (2013) Diet composition and activity level of at risk and metabolically healthy obese American adults. Obesity (Silver Spring) 21, 637–643. 23592673 10.1002/oby.20257 PMC3416914

[7] Kant AK (2004) Dietary patterns and health outcomes. J Am Diet Assoc 104, 615–635. 15054348 10.1016/j.jada.2004.01.010

[8] Einsele F, Sadeghi L, Ingold R & Jenzer H (2105) A study about discovery of critical food consumption patterns linked with lifestyle diseases using data mining methods. Proc Int Conf Health Inf (HEALTHINF-2015), 1, 239–245.

[9] Marques-Vidal P, Waeber G, Vollenweider P & Guessous I (2018) Socio-demographic and lifestyle determinants of dietary patterns in French-speaking Switzerland, 2009–2012. BMC Public Health 18, 131. 29329572 10.1186/s12889-018-5045-1 PMC5766995

[10] Jessri M, Ng AP & L'Abbé MR (2017) Adapting the healthy eating index 2010 for the Canadian population: evidence from the Canadian National Nutrition Survey. Nutrients 9, 910. 10.3390/nu9080910 PMC5579703 28825674

[11] World Health Organization (2014) Global Status report on noncommunicable diseases 2014. Geneva, Switzerland: WHO. https://www.who.int/nmh/publications/ncd-status-report-2014/en/ (accessed November 2019).

[12] World Health Organization (2017) Noncommunicable Diseases Progress Monitor. Geneva: World Health Organization 2017. License: CC BY-NC-SA 3⋅0 (accessed October 2019).

[13] Schwingshackl L, Bogensberger B & Hoffmann G (2018) Diet quality as assessed by the healthy eating index, alternate healthy eating index, dietary approaches to stop hypertension score, and health outcomes: an updated systematic review and meta-analysis of cohort studies. J Acad Nutr Diet 118, 74–100. e11. 29111090 10.1016/j.jand.2017.08.024

[14] Naja F, Shatila H, Meho L (2017) Gaps and opportunities for nutrition research in relation to non-communicable diseases in Arab countries: call for an informed research agenda. Nutr Res 47, 1–12. 29241573 10.1016/j.nutres.2017.07.011

[15] Hoffmann I (2003) Transcending reductionism in nutrition research. Am J Clin Nutr 78, 514S–516S. 12936942 10.1093/ajcn/78.3.514S

[16] Oguoma VM, Nwose EU, Skinner TC (2018) Diet and lifestyle habits: association with cardiovascular disease indices in a Nigerian sub-population. Diabetes Metab Syndr 12, 653–659. 29673925 10.1016/j.dsx.2018.04.007

[17] Shim JS, Oh K & Kim HC (2014) Dietary assessment methods in epidemiologic studies. Epidemiol Health 36, e2014009. 25078382 10.4178/epih/e2014009 PMC4154347

[18] Rodrigo CP, Aranceta J, Salvador G (2015) Food frequency questionnaires. Nutr Hosp 31, Suppl. 3, 49–56. 25719771 10.3305/nh.2015.31.sup3.8751

[19] Gkza A & Davenport A (2017) Estimated dietary sodium intake in haemodialysis patients using food frequency questionnaires. Clin Kidney J 10, 715–720. 28979785 10.1093/ckj/sfx037 PMC5622899

[20] Yu L, Liu L, Wang F (2019) Higher frequency of dairy intake is associated with a reduced risk of breast cancer: results from a case-control study in Northern and Eastern China. Oncol Lett 17, 2737–2744. 30854047 10.3892/ol.2019.9898 PMC6365923

[21] Archundia Herrera MC, Subhan FB & Chan CB (2017) Dietary patterns and cardiovascular disease risk in people. Curr Obes Rep 6, 405–413. 29063379 10.1007/s13679-017-0284-5

[22] Panagiotakos DB, Notara V, Kouvari M (2016) The Mediterranean and other dietary patterns in secondary cardiovascular disease prevention: a review. Curr Vasc Pharmacol 14, 442–451. 27456104 10.2174/1570161114999160719104731

[23] Dragsted LO (2017) Relying on biomarkers for intake assessment in nutrition. Am J Clin Nutr 105, 8–9. 28003209 10.3945/ajcn.116.148320 PMC5183736

[24] Sébédio JL (2017) Metabolomics, nutrition, and potential biomarkers of food quality, intake, and health Status. Adv Food Nutr Res 82, 83–116. 28427537 10.1016/bs.afnr.2017.01.001

[25] Corella D & Ordovas JM. (2015) Biomarkers: background, classification and guidelines for applications in nutritional epidemiology. Nutr Hosp 31, Suppl. 3, 177–188. 25719785 10.3305/nh.2015.31.sup3.8765

[26] Kaaks R, Ferrari P, Ciampi A (2002) Uses and limitations of statistical accounting for random error correlations, in the validation of dietary questionnaire assessments. Public Health Nutr 5, 969–976. 12638598 10.1079/phn2002380

[27] Potischman N (2003) Biologic and methodologic issues for nutritional biomarkers. J Nutr 133, Suppl. 3, 875S–880S. 12612173 10.1093/jn/133.3.875S

[28] Pachucki MA (2012) Food pattern analysis over time: unhealthful eating trajectories predict obesity. Int J Obes *(*Lond) 36, 686–694. 21792169 10.1038/ijo.2011.133 PMC3212637

[29] Thompson FE, Kirkpatrick SI, Subar AF (2015) The National Cancer Institute's dietary assessment primer: a resource for diet research. J Acad Nutr Diet, 115, 1986–1995. 26422452 10.1016/j.jand.2015.08.016 PMC4663113

[30] Moeller SM, Reedy J, Millen AE (2007) Dietary patterns: challenges and opportunities in dietary patterns research an experimental biology workshop, April 1, 2006. J Am Diet Assoc 107, 1233–1239. 17604756 10.1016/j.jada.2007.03.014

[31] Schulze MB, Martínez-González MA, Fung TT (2018) Food based dietary patterns and chronic disease prevention. Br Med J 361, k2396. 29898951 10.1136/bmj.k2396 PMC5996879

[32] Hu FB (2002) Dietary pattern analysis: a new direction in nutritional epidemiology. Curr Opin Lipidol 13, 3–9. 11790957 10.1097/00041433-200202000-00002

[33] Gil A, Martinez de Victoria E & Olza J (2015) Indicators for the evaluation of diet quality. Nutr Hosp 31, Suppl. 3, 128–144. 25719781 10.3305/nh.2015.31.sup3.8761

[34] Jennings A, Welch A, van Sluijs EM, (2011) Diet quality is independently associated with weight status in children aged 9–10 years. J Nutr 141, 453–459. 21270356 10.3945/jn.110.131441

[35] Venkaiah K, Brahman GNV & Vijayaraghavan K (2011) Application of factor analysis to identify dietary patterns and use of factor scores to study their relationship with nutritional status of adult rural populations. J Health Popul Nutr 29, 327–338. 21957671 10.3329/jhpn.v29i4.8448 PMC3190363

[36] Shroff MR, Perng W, Baylin A (2014) Adherence to a snacking dietary pattern and soda intake are related to the development of adiposity: a prospective study in school-age children. Public Health Nutr 17, 1507–1513. 23701749 10.1017/S136898001300133X PMC10282458

[37] Voortman T, Leermakers ET, Franco OH (2016) A priori and a posteriori dietary patterns at the age of 1 year and body composition at the age of 6 years: the Generation R Study. Eur J Epidemiol 31, 775–783. 27384175 10.1007/s10654-016-0179-x PMC5005385

[38] Weikert C & Schulze MB (2016) Evaluating dietary patterns: the role of reduced rank regression. Curr Opin Clin Nutr Metab Care 19, 341–346. 27389081 10.1097/MCO.0000000000000308

[39] Trichopoulos D & Lagiou P (2001) Dietary patterns and mortality. Br J Nutr 85, 133–134. 11242479 10.1079/bjn2000282

[40] Ocke MC (2013) Evaluation of methodologies for assessing the overall diet: dietary quality scores and dietary pattern analysis. Proc Nutr Soc 72, 191–199. 23360896 10.1017/S0029665113000013

[41] Hoffmann K, Zyriax BC, Boeing H (2004) A dietary pattern derived to explain biomarker variation is strongly associated with the risk of coronary artery disease. Am J Clin Nutr 80, 633–640. 15321803 10.1093/ajcn/80.3.633

[42] Gibson RS, Charrondiere UR & Bell W (2017) Measurement errors in dietary assessment using self-reported 24-hour recalls in low-income countries and strategies for their prevention. Adv Nutr 8, 980–991. 29141979 10.3945/an.117.016980 PMC5683000

[43] Waijers PM, Feskens EJ & Ocké MC (2007) A critical review of predefined diet quality scores. Br J Nutr 97, 219–231. 17298689 10.1017/S0007114507250421

[44] Román-Viñas B, Ribas Barba L, Ngo J (2009) Validity of dietary patterns to assess nutrient intake adequacy. Br J Nutr 101, Suppl. 2, S12–S20. 19594960 10.1017/S0007114509990547

[45] Haines PS, Siega-Riz AM & Popkin BM (1999) The diet quality index revised: a measurement instrument for populations. J Am Diet Assoc 99, 697–704. 10361532 10.1016/S0002-8223(99)00168-6

[46] Wirfält E, Drake I & Wallström P (2013) What do review papers conclude about food and dietary patterns?. Food Nutr Res [Epublication 4 March 2013]. 10.3402/fnr.v57i0.20523 PMC3589439 23467387

[47] Azadbakht L & Esmaillzadeh A (2009) Diet variety: a measure of nutritional adequacy and health. J Qazvin Univ Med Sci 13, 88–97.

[48] Nachvak SM, Abdollahzad H, Mostafai R (2017) Dietary diversity score and its related factors among employees of Kermanshah University of Medical Sciences. Clin Nutr Res 6, 247–255. 29124045 10.7762/cnr.2017.6.4.247 PMC5665746

[49] Kranz S & McCabe GP. Examination of the five comparable component scores of the diet quality indexes HEI-2005 and RC-DQI using a nationally representative sample of 2–18 years old children: NHANES 2003–2006. J Obes [Epublication 15 September 2013]. 10.1155/2013/376314 PMC3791824 24163762

[50] Trichopoulou A, Costacou T, Bamia C (2003) Adherence to a Mediterranean diet and survival in a Greek population. N Engl J Med 348, 2599–2608. 12826634 10.1056/NEJMoa025039

[51] Kennedy ET, Ohls J, Carlson S (1995) The healthy eating index: design and applications. J Am Diet Assoc 95, 1103-1108. 7560680 10.1016/S0002-8223(95)00300-2

[52] Reedy J, Lerman JL, Krebs-Smith SM (2018) Evaluation of the healthy eating index-2015. J Acad Nutr Diet 118, 1622–1633. 30146073 10.1016/j.jand.2018.05.019 PMC6718954

[53] Olza J, Martínez de Victoria E, Aranceta-Bartrina J (2019) Adequacy of critical nutrients affecting the quality of the Spanish diet in the ANIBES study. Nutrients 11, 2328. 10.3390/nu11102328 PMC6835880 31581518

[54] Martinez ME, Marshall JR & Sechrest L (1998) Invited commentary: factor analysis and the search for objectivity. Am J Epidemiol 148, 17–19. 9663398 10.1093/oxfordjournals.aje.a009552

[55] Paradis AM, Pérusse L & Vohl MC (2006) Dietary patterns and associated lifestyles in individuals with and without familial history of obesity: a cross-sectional study. Int J Behav Nutr Phys Act 3, 38. 17076904 10.1186/1479-5868-3-38 PMC1635721

[56] Tucker KL (2010) Dietary patterns, approaches, and multicultural perspective. Appl Physiol Nutr Metabol 35, 211–218. 10.1139/H10-010 20383235

[57] Niu K, Momma H, Kobayashi Y (2016) The traditional Japanese dietary pattern and longitudinal changes in cardiovascular disease risk factors in apparently healthy Japanese adults. Eur J Nutr 55, 267–279. 25648737 10.1007/s00394-015-0844-y

[58] Drehmer M, Odegaard AO, Schmidt MI (2017) Brazilian dietary patterns and the dietary approaches to stop hypertension (DASH) diet-relationship with metabolic syndrome and newly diagnosed diabetes in the ELSA-Brasil study. Diabetol Metab Syndr 13, 9, 13. 10.1186/s13098-017-0211-7 PMC5307839 28228848

[59] Hoffmann K, Schulze MB, Schienkiewitz A (2004) Application of a new statistical method to derive dietary patterns in nutritional epidemiology. Am J Epidemiol 159, 935–944. 15128605 10.1093/aje/kwh134

[60] van Dam RM (2005) New approaches to the study of dietary patterns. Br J Nutr 93, 573–574. 15975154 10.1079/bjn20051453

[61] Hoffman K, Schulze MB, Boeing H (2002) Dietary patterns: report of an international workshop. Public Health Nutr 5, 89–90. 12001983 10.1079/phn2001252

[62] Cunha DB, Sichieri R, de Almeida RMVR (2011) Factors associated with dietary patterns among low-income adults. Public Health Nutr 14, 1579–1585. 21241535 10.1017/S136898001000354X

[63] Jacques PF & Tucker KL (2001) Are dietary patterns useful for understanding the role of diet in chronic disease?. Am J Clin Nutr 73, 1–2. 11124739 10.1093/ajcn/73.1.1

[64] Perozzo G, Olinto MT, Dias-da-Costa JS (2008) Association between dietary patterns and body mass index and waist circumference in women living in southern Brazil. Cad Saude Publica 24, 2427–2439. 18949244 10.1590/s0102-311x2008001000023

[65] Cai H, Zheng W, Xiang YB (2007) Dietary patterns and their correlates among middle-aged and elderly Chinese men: a report from the Shanghai Men's Health Study. Br J Nutr 98, 1006–1013. 17524168 10.1017/S0007114507750900

[66] Krieger JP, Pestoni G, Cabaset S (2018) Dietary patterns and their sociodemographic and lifestyle determinants in Switzerland: results from the National Nutrition Survey menu CH. Nutrients 11, pii:E62. 10.3390/nu11010062 PMC6356790 30597962

[67] Fransen HP, Boer JMA, Beulens JWJ (2017) Associations between lifestyle factors and an unhealthy diet. Eur J Public Health 27, 274–278. 27744349 10.1093/eurpub/ckw190

[68] Noble NE, Paul CL, Turner N (2015) A cross-sectional survey and latent class analysis of the prevalence and clustering of health risk factors among people attending an Aboriginal Community Controlled Health Service. BMC Public Health 15, 666. 26173908 10.1186/s12889-015-2015-8 PMC4502927

[69] Akbarpour S, Khalili D, Zeraati H (2018) Lifestyle patterns in the Iranian population: self- organizing map application. Caspian J Intern Med 9, 268–275. 30197772 10.22088/cjim.9.3.268 PMC6121339

[70] Kesse-Guyot E, Bertrais S, Péneau S (2009) Dietary patterns and their sociodemographic and behavioural correlates in French middle-aged adults from the SU.VI.MAX cohort. Eur J Clin Nutr 63, 521–528. 18212801 10.1038/sj.ejcn.1602978

[71] Patino-Alonso MC, Recio-Rodriguez JI, Belio JF (2014) Factors associated with adherence to the Mediterranean diet in the adult population. J Acad Nutr Diet 114, 583–589. 24209889 10.1016/j.jand.2013.07.038

[72] Li Y, Pan A, Wang DD (2018) Impact of healthy lifestyle factors on life expectancies in the US population. Circulation 138, 345–355. 29712712 10.1161/CIRCULATIONAHA.117.032047 PMC6207481

[73] Schwingshackl L, Schwedhelm C, Hoffmann G (2017) Food groups and risk of all-cause mortality: a systematic review and meta-analysis of prospective studies. Am J Clin Nutr 105, 1462–1473. 28446499 10.3945/ajcn.117.153148

[74] Xiao Q, Keadle SK, Hollenbeck AR (2014) Sleep duration and total and cause-specific mortality in a large US cohort: interrelationships with physical activity, sedentary behavior, and body mass index. Am J Epidemiol 180, 997–1006. 25281691 10.1093/aje/kwu222 PMC4224361

[75] Oftedal S, Vandelanotte C & Duncan MJ (2019) Patterns of diet, physical activity, sitting and sleep are associated with socio-demographic, behavioural, and health-risk indicators in adults. Int J Environ Res Public Health 16, 2375. 10.3390/ijerph16132375 PMC6651368 31277386

[76] Knudsen VK, Matthiessen J, Biltoft-Jensen A (2014) Identifying dietary patterns and associated health-related lifestyle factors in the adult Danish population. Eur J Clin Nutr 68, 736–740. 24642781 10.1038/ejcn.2014.38

[77] Arruda SP, da Silva AA, Kac G (2014) Socioeconomic and demographic factors are associated with dietary patterns in a cohort of young Brazilian adults. BMC Public Health 14, 654. 24969831 10.1186/1471-2458-14-654 PMC4082487

[78] Schneider S, Huy C, Schuessler M (2009) Optimising lifestyle interventions: identification of health behaviour patterns by cluster analysis in a German 50+ survey. Eur J Public Health 19, 271–277. 19164433 10.1093/eurpub/ckn144

[79] Kiefte-de Jong JC, de Vries JH, Bleeker SE (2013) Socio-demographic and lifestyle determinants of ‘Western-like’ and ‘Health conscious’ dietary patterns in toddlers. J Nutr 109, 137–147. 10.1017/S0007114512000682 22475342

[80] Heppe DH, Kiefte-de Jong J, Durmus B (2013) Parental, fetal, and infant risk factors for preschool overweight: the Generation R Study. Pediatr Res 73, 120–127. 23138398 10.1038/pr.2012.145

[81] Lobo AS, de Assis MAA, Leal DB (2019) Empirically derived dietary patterns through latent profile analysis among Brazilian children and adolescents from Southern Brazil, 2013-2015. PLoS ONE 14, e0210425. 30620755 10.1371/journal.pone.0210425 PMC6324812

[82] Craigie AM, Lake AA, Kelly SA (2011) Tracking of obesity-related behaviors from childhood to adulthood: a systematic review. Maturitas 70, 266–284. 21920682 10.1016/j.maturitas.2011.08.005

[83] Lobstein T, Jackson-Leach R, Moodie ML (2015) Child and adolescent obesity: part of a bigger picture. Lancet 385, 2510–2520. 25703114 10.1016/S0140-6736(14)61746-3 PMC4594797

[84] Costa FF, Schmoelz CP, Davies VF (2013) Assessment of diet and physical activity of Brazilian schoolchildren: usability testing of a web-based questionnaire. JMIR Res Protoc 19, e31. 10.2196/resprot.2646 PMC3758065 23958804

[85] McCarthy S (2014) Weekly patterns, diet quality and energy balance. Physiol Behav 134, 55–59. 24589367 10.1016/j.physbeh.2014.02.046

[86] Svensson A, Larsson C, Eiben G (2014) European children's sugar intake on weekdays versus weekends: the IDEFICS study. Eur J Clin Nutr 68, 822–828. 24824016 10.1038/ejcn.2014.87

[87] Rothausen BW, Matthiessen J, Andersen LF (2013) Dietary patterns on weekdays and weekend days in 4–14-year-old Danish children. Br J Nutr 109, 1704–1713. 22958341 10.1017/S0007114512003662

[88] Leech RM, McNaughton SA & Timperio A (2014) The clustering of diet, physical activity and sedentary behavior in children and adolescents: a review. Int J Behav Nutr Phys Act 11, 4. 24450617 10.1186/1479-5868-11-4 PMC3904164

[89] Dayana M, Reshma M & Dhanalekshmy TG (2019) A random study of dietary pattern and nutritional knowledge among adolescent girls in an urban area. Int J Res Pharm Biosci 6, 1–7.

[90] Omage K & Omuemu VO (2018) Assessment of dietary pattern and nutritional status of undergraduate students in a private university in southern Nigeria. Food Sci Nutr 6, 1890–1897. 30349678 10.1002/fsn3.759 PMC6189614

[91] Marques-Vidal P, Bovet P, Paccaud F (2010) Changes of overweight and obesity in the adult Swiss population according to educational level, from 1992 to 2007. BMC Public Health 10, 87. 20170554 10.1186/1471-2458-10-87 PMC2831837

[92] Fung TT, Schulze M, Manson JE (2004) Dietary patterns, meat intake, and the risk of type 2 diabetes in women. Arch Intern Med 164, 2235–2240. 15534160 10.1001/archinte.164.20.2235

[93] Oliveira A, Rodríguez-Artalejo F, Gaio R (2011) Major habitual dietary patterns are associated with acute myocardial infarction and cardiovascular risk markers in a southern European population. J Am Diet Assoc 111, 241–250. 21272698 10.1016/j.jada.2010.10.042

[94] Reininger B, Lee M, Jennings R (2016) Healthy eating patterns associated with acculturation, sex and BMI among Mexican Americans. Public Health Nutr 20, 1267–1278. 28004615 10.1017/S1368980016003311 PMC10261606

[95] Imoscopi A, Inelmen EM, Sergi G (2012) Taste loss in the elderly: epidemiology, causes and consequences. Aging Clin Exp Res 24, 570–579. 22828477 10.3275/8520

[96] Drewnowski A (2010) The cost of US foods as related to their nutritive value. Am J Clin Nutr 92, 1181–1188. 20720258 10.3945/ajcn.2010.29300 PMC2954450

[97] Lopez-Garcia E, Schulze MB, Fung TT (2004) Major dietary patterns are related to plasma concentrations of markers of inflammation and endothelial dysfunction. Am J Clin Nutr 80, 1029–1035. 15447916 10.1093/ajcn/80.4.1029

[98] Everitt AV, Hilmer SN, Brand-Miller JC (2006) Dietary approaches that delay age-related diseases. Clin Interv Aging 1, 11–31. 18047254 10.2147/ciia.2006.1.1.11 PMC2682451

[99] Zhao W, Ukawa S & Okada E (2019) The associations of dietary patterns with all-cause mortality and other lifestyle factors in the elderly: an age-specific prospective cohort study. Clin Nutr 38, 288–296. 29428786 10.1016/j.clnu.2018.01.018

[100] Si Hassen W, Castetbon K, Cardon P (2016) Socioeconomic indicators are independently associated with nutrient intake in French adults: a DEDIPAC study. Nutrients 8, 158. 26978393 10.3390/nu8030158 PMC4808886

[101] Boylan S, Lallukka T, Lahelma E (2011) Socio-economic circumstances and food habits in Eastern, Central and Western European populations. Public Health Nutr 14, 678–687. 20843403 10.1017/S1368980010002570 PMC3951866

[102] Mayen AL, Marques-Vidal P, Paccaud F (2014) Socioeconomic determinants of dietary patterns in low- and middle-income countries: a systematic review. Am J Clin Nutr 100, 1520–1531. 25411287 10.3945/ajcn.114.089029

[103] Lutomski JE, van den Broeck J, Harrington J, (2011) Sociodemographic, lifestyle, mental health and dietary factors associated with direction of misreporting of energy intake. Public Health Nutr 14, 532–541. 20707944 10.1017/S1368980010001801

[104] de Abreu D, Guessous I, Vaucher J (2013) Low compliance with dietary recommendations for food intake among adults. Clin Nutr 32, 783–788. 23260749 10.1016/j.clnu.2012.11.022

[105] Lenz A, Olinto MT, Dias-da-Costa JS (2009) Socioeconomic, demographic and lifestyle factors associated with dietary patterns of women living in southern Brazil. Cad Saude Publica 25, 1297–1306. 19503960 10.1590/s0102-311x2009000600012

[106] Peltzer K & Pengpid S (2015) Correlates of healthy fruit and vegetables diet in students in low, middle and high income countries. Int J Public Health 60, 79–90. 25504430 10.1007/s00038-014-0631-1

[107] Monteiro LZ, Varela AR, Lira Bade (2019) Weight status, physical activity and eating habits of young adults in Midwest Brazil. Public Health Nutr 22, 2609–2616. 31148525 10.1017/S1368980019000995 PMC10260673

[108] Khabaz MN, Bakarman MA, Baig M (2017) Dietary habits, lifestyle pattern and obesity among young Saudi university students. J Pak Med Assoc 67, 1541–1546. 28955071

[109] Amuna P & Zotor FB (2008) Epidemiological and nutrition transition in developing countries: impact on human health and development: the epidemiological and nutrition transition in developing countries: evolving trends and their impact in public health and human development. Proc Nutr Soc 67, 82–90. 18234135 10.1017/S0029665108006058

[110] Suh SY, Lee JH, Park SS (2013) Less healthy dietary pattern is associated with smoking in Korean men according to nationally representative data. J Korean Med Sci 28, 869–875. 23772151 10.3346/jkms.2013.28.6.869 PMC3678003

[111] Northrop-Clewes CA & Thurnham DI (2007) Monitoring micronutrients in cigarette smokers. Clin Chim Acta 377, 14–38. 17045981 10.1016/j.cca.2006.08.028

[112] Northstone K & Emmett PM (2010) Dietary patterns of men in ALSPAC: associations with socio-demographic and lifestyle characteristics, nutrient intake and comparison with women's dietary patterns. Eur J Clin Nutr 64, 978–986. 20571501 10.1038/ejcn.2010.102

[113] Yamauchi Y, Endo S & Yoshimura I (2002) A new whole-mouth gustatory test procedure. II. Effects of aging, gender and smoking. Acta Otolaryngol Suppl 546, 49–59. 10.1080/00016480260046418 12132621

[114] Vennemann MM, Hummel T & Berger K (2008) The association between smoking and smell and taste impairment in the general population. J Neurol 255, 1121–1126. 18677645 10.1007/s00415-008-0807-9

[115] Hu EA, Toledo E, Diez-Espino J (2013) Lifestyles and risk factors associated with adherence to the Mediterranean diet: a baseline assessment of the PREDIMED trial. PLoS ONE 8, e60166. 23637743 10.1371/journal.pone.0060166 PMC3639284

[116] Hernandez-Hernandez A, Gea A, Ruiz-Canela M (2015) Mediterranean alcohol-drinking pattern and the incidence of cardiovascular disease and cardiovascular mortality: the SUN project. Nutrients 7, 9116–9126. 26556367 10.3390/nu7115456 PMC4663584

[117] Estruch R, Ros E, Salas-Salvadó J (2013) Primary prevention of cardiovascular disease with a Mediterranean diet. N Engl J Med 368, 1279–1290. 23432189 10.1056/NEJMoa1200303

[118] Barefoot JC, Grønbaek M, Feaganes JR (2002) Alcoholic beverage preference, diet, and health habits in the UNC alumni heart study. Am J Clin Nutr 76, 466–472. 12145024 10.1093/ajcn/76.2.466

[119] Reynolds K, Lewis B, Nolen JD (2003) Alcohol consumption and risk of stroke: a meta-analysis. JAMA 289, 579–588. 12578491 10.1001/jama.289.5.579

[120] Laatikainen T, Manninen L, Poikolainen K (2003) Increased mortality related to heavy alcohol intake pattern. J Epidemiol Community Health 57, 379–384. 12700224 10.1136/jech.57.5.379 PMC1732462

[121] Berg CM, Lappas G, Strandhagen E (2008) Food patterns and cardiovascular disease risk factors: the Swedish INTERGENE research program. Am J Clin Nutr 88, 289–297. 18689363 10.1093/ajcn/88.2.289

[122] Charreire H, Kesse-Guyot E, Bertrais S (2011) Associations between dietary patterns, physical activity (leisure-time and occupational) and television viewing in middle-aged French adults. Br J Nutr 105, 902–910. 21251337 10.1017/S000711451000440X

[123] Mesas AE, Leon-Munoz LM, Guallar-Castillon P (2012) Obesity related eating behaviours in the adult population of Spain, 2008–2010. Obes Rev 13, 858–867. 22577840 10.1111/j.1467-789X.2012.01005.x

[124] Esmaillzadeh A & Azadbakht L (2008) Major dietary patterns in relation to general obesity and central adiposity among Iranian women. J Nutr 138, 358–363. 18203904 10.1093/jn/138.2.358

[125] Jezewska-Zychowicz M, Gębski J, Plichta M (2019) Diet-related factors, physical activity, and weight status in polish adults. Nutrients 11, 2532. 10.3390/nu11102532 PMC6835331 31640114

[126] Iftikhar IH, Donley MA, Mindel J (2015) Sleep duration and metabolic syndrome. An updated dose-risk metaanalysis. Ann Am Thorac Soc 12, 1364–1372. 26168016 10.1513/AnnalsATS.201504-190OC PMC5467093

[127] Koren D, O'Sullivan KL & Mokhlesi B (2015) Metabolic and glycemic sequelae of sleep disturbances in children and adults. Curr Diab Rep 15, 562. 25398202 10.1007/s11892-014-0562-5 PMC4467532

[128] Pot GK (2018) Sleep and dietary habits in the urban environment: the role of chrono-nutrition. Proc Nutr Soc 77, 189–198. 29065932 10.1017/S0029665117003974

[129] Grandner MA, Jackson N, Gerstner JR (2013) Dietary nutrients associated with short and long sleep duration. Data from a nationally representative sample. Appetite 64, 71–80. 23339991 10.1016/j.appet.2013.01.004 PMC3703747

[130] Beydoun MA, Gamaldo AA, Canas JA (2014) Serum nutritional biomarkers and their associations with sleep among US adults in recent national surveys. PLoS ONE 9, e103490. 25137304 10.1371/journal.pone.0103490 PMC4138077

[131] Mondin TC, Stuart AL, Williams LJ (2019) Diet quality, dietary patterns and short sleep duration: a cross-sectional population-based study. Eur J Nutr 58, 641–651. 29516221 10.1007/s00394-018-1655-8

[132] Marshall NS, Glozier N & Grunstein RR (2008) Is sleep duration related to obesity? A critical review of the epidemiological evidence. Sleep Med Rev 12, 289–298. 18485764 10.1016/j.smrv.2008.03.001

[133] Almoosawi S, Palla L, Walshe I (2018) Long sleep duration and social jetlag are associated inversely with a healthy dietary pattern in adults: results from the UK national diet and nutrition survey rolling programme Y1-4. Nutrients 10, pii: E1131. 10.3390/nu10091131 PMC6163907 30134567

[134] Koball HL, Moiduddin E, Henderson J (2010) What do we know about the link between marriage and health? J Fam Issues 31, 1019–1040.

[135] Bowen L, Ebrahim S, De Stavola B (2011) Dietary intake and rural-urban migration in India: a cross-sectional study. PLoS ONE 6, e14822. 21731604 10.1371/journal.pone.0014822 PMC3120774

[136] Malon A, Deschamps V, Salanave B (2010) Compliance with French nutrition and health program recommendations is strongly associated with socioeconomic characteristics in the general adult population. J Am Diet Assoc 110, 848–856. 20497773 10.1016/j.jada.2010.03.027

[137] Tielemans MJ, Erler NS, Leermakers ET (2015) A priori and a posteriori dietary patterns during pregnancy and gestational weight gain: the Generation R Study. Nutrients 7, 9383–9399. 26569303 10.3390/nu7115476 PMC4663604

[138] Keen CL, Clegg MS, Hanna LA (2003) The plausibility of micronutrient deficiencies being a significant contributing factor to the occurrence of pregnancy complications. J Nutr 133, Suppl. 2, S1597–S1605. 10.1093/jn/133.5.1597S 12730474

[139] Uusitalo U, Arkkola T, Ovaskainen ML (2009) Unhealthy dietary patterns are associated with weight gain during pregnancy among Finnish women. Public Health Nutr 12, 2392–2399. 19323867 10.1017/S136898000900528X

[140] Cuco G, Fernandez-Ballart J, Sala J (2006) Dietary patterns and associated lifestyles in preconception, pregnancy and postpartum. Eur J Clin Nutr 60, 364–371. 16340954 10.1038/sj.ejcn.1602324

[141] Leech RM, Worsley A, Timperio A (2015) Characterizing eating patterns: a comparison of eating occasion definitions. Am J Clin Nutr 102, 1229–1237. 26447152 10.3945/ajcn.115.114660

[142] Leech RM, Worsley A, Timperio A (2015) Understanding meal patterns: definitions, methodology and impact on nutrient intake and diet quality. Nutr Res Rev 28, 1–21. 25790334 10.1017/S0954422414000262 PMC4501369

[143] Min C, Noh H, Kang YS (2011) Skipping breakfast is associated with diet quality and metabolic syndrome risk factors of adults. Nutr Res Pract 5, 455–463. 22125684 10.4162/nrp.2011.5.5.455 PMC3221832

[144] Mekary RA, Giovannucci E, Cahill L (2013) Eating patterns and type 2 diabetes risk in older women: breakfast consumption and eating frequency. Am J Clin Nutr 98, 436–443. 23761483 10.3945/ajcn.112.057521 PMC3712552

[145] Chatelan A, Castetbon K, Pasquier J (2018) Association between breakfast composition and abdominal obesity in the Swiss adult population eating breakfast regularly. Int J Behav Nutr Phys Act 15, 115. 30458811 10.1186/s12966-018-0752-7 PMC6247634

[146] Deshmukh-Taskar PR, Radcliffe JD, Liu Y (2010) Do breakfast skipping and breakfast type affect energy intake, nutrient intake, nutrient adequacy, and diet quality in young adults? NHANES 1999–2002. J Am Coll Nutr 29, 407–418. 21041816 10.1080/07315724.2010.10719858

[147] O'Neil CE, Nicklas TA & Fulgoni VL III (2014) Nutrient intake, diet quality, and weight/adiposity parameters in breakfast patterns compared with no breakfast in adults: national health and nutrition examination survey 2001–2008. J Acad Nutr Diet 114, Suppl. 12, S27–S43. 25458992 10.1016/j.jand.2014.08.021

[148] Odegaard AO, Jacobs DR Jr, Steffen LM (2013) Breakfast frequency and development of metabolic risk. Diabetes Care 36, 3100–3106. 23775814 10.2337/dc13-0316 PMC3781522

[149] St Onge MP, Ard J, Baskin ML (2017) Meal timing and frequency: implications for cardiovascular disease prevention: a scientific statement from the American Heart Association. Circulation 135, e96–e121. 28137935 10.1161/CIR.0000000000000476 PMC8532518

[150] Juan S, He Y, Zhiyue L (2013). Factors associated with skipping breakfast among Inner Mongolia medical students in China. BMC Public Health 13, 42. 23327195 10.1186/1471-2458-13-42 PMC3562148

[151] Kutsuma A, Nakajima K & Suwa K. Potential association between breakfast skipping and concomitant late-night-dinner eating with metabolic syndrome and proteinuria in the Japanese population. Scientifica *(*Cairo*)* [Epublication 25 March 2014]. 10.1155/2014/253581 PMC3984843 24982814

[152] Jun YS, Choi MK & Bae YJ (2015). Night eating and nutrient intake status according to residence type in university students. J Korean Soc Food Sci Nutr 44, 216–225.

[153] Striegel-Moore RH, Franko DL, Thompson D (2006) Night eating: prevalence and demographic correlates. Obesity *(*Silver Spring*)* 14, 139–147. 16493132 10.1038/oby.2006.17

[154] Lucassen EA, Zhao X, Rother KI (2013) Evening chronotype is associated with changes in eating behavior, more sleep apnea, and increased stress hormones in short sleeping obese individuals. PLoS ONE 8, e56519. 23483886 10.1371/journal.pone.0056519 PMC3590198

[155] Sato-Mito N, Shibata S, Sasaki S (2011) Dietary intake is associated with human chronotype as assessed by morningness–eveningness score and preferred midpoint of sleep in young Japanese women. Int J Food Sci Nutr 62, 525–532. 21495902 10.3109/09637486.2011.560563

[156] Baron KG, Reid KJ, Kern AS (2011) Role of sleep timing in caloric intake and BMI. Obesity *(*Silver Spring*)* 19, 1374–1381. 21527892 10.1038/oby.2011.100

[157] Berg C, Lappas G, Wolk A (2009) Eating patterns and portion size associated with obesity in a Swedish population. Appetite 52, 21–26. 18694791 10.1016/j.appet.2008.07.008

[158] Harb A, Levandovski R, Oliveira C (2012) Night eating patterns and chronotypes: a correlation with binge eating behaviors. Psychiatry Res 200, 489–493. 22906954 10.1016/j.psychres.2012.07.004

[159] Lachat C, Nago E, Verstraeten R (2012) Eating out of home and its association with dietary intake: a systematic review of the evidence. Obes Rev 13, 329–346. 22106948 10.1111/j.1467-789X.2011.00953.x

[160] Nicklas TA, Baranowski T, Cullen KW (2001) Eating patterns, dietary quality and obesity. J Am Coll Nutr 20, 599–608. 11771675 10.1080/07315724.2001.10719064

[161] Zong G, Eisenberg DM, Hu FB (2016) Consumption of meals prepared at home and risk of type 2 diabetes: an analysis of two prospective cohort studies. PLoS Med 13, e1002052. 27379673 10.1371/journal.pmed.1002052 PMC4933392

[162] Wolfson JA & Bleich SN (2015) Is cooking at home associated with better diet quality or weight-loss intention?. Public Health Nutr 18, 1397–1406. 25399031 10.1017/S1368980014001943 PMC8728746

